# Psychology of Berkeley
*"Works of George Berkeley, D.D., Bishop of Cloyne."


**Published:** 1855-07-01

**Authors:** 


### Art IV.—PSYCHOLOGY OF BERKELEY.*

Six or seven years ago, a book was published to prove that there is no existence but *mind* or *spirit* in the universe, all the supposed materialism around us being only an illusive and unreal phantom. A prize of one hundred pounds was offered to any one who, in the judgment of some three or four individuals agreed on by the author and the respondent as competent to decide, should be pronounced to have satisfactorily refuted the arguments of the former. This challenge was never publicly heard of more, and therefore we conclude was never accepted. To the uninitiated in the history of metaphysics, the above fact may seem curious enough; but it may serve at all events to show that the Berkeleian cosmology and psychology (which are one) exhibit a phase of speculation which, however strange and staggering its results, has something to say for itself which is too plausible or perplexing to be answered off-hand—either to be refuted by Dr. Johnson stamping with his foot, or as Pope has it,—

> "To be vanquished by coxcombs with a grin."

We have little space for Berkeley's history; but the purity, benevolence, and disinterestedness of his character, in connexion with his extraordinary talents, gained him deserved admiration in his day Pope ascribed "to Berkeley every virtue under heaven;" and Atterbury, an acute but not very charitably-tempered man, said, after his first interview with him: "so much understanding, so much knowledge, so much innocence, and such humility, I did not think had been the portion of any but angels, till I saw this gentleman." Adverse factions and hostile wits, as Sir James Macintosh remarks, concurred "in loving, admiring, and contributing to advance him." He was born in Kilkenny, in 1684. In 1709, appeared his "New Theory of Vision;" and the next year his "Principles of Human Knowledge," in which he totally denies the existence of every kind of matter, whatever, independently of the phenomena of mind. In 1712, he defended still further his system of Immaterialism, in his "Three Dialogues between Hylas and Philonous." He wrote a number of other works, but the above contain his metaphysical theories. His "Minute Philosopher" was addressed to the various characters which the free-thinking of the times had assumed; and his "Analyst," and his "Defence of Free-thinking in Mathematics," were designed to prove that mathematicians admitted mysteries into science greater than those of faith; and that the doctrine of fluxions comprised even

* "Works of George Berkeley, D.D., Bishop of Cloyne."

falsehoods. The latter allegation gave rise to Robins's "Discourse," and Maclaurin's "Treatise on Fluxions," and was thus satisfactorily answered.

One of the most remarkable circumstances in the life of Berkeley was his offering to resign the deanery of Derry, worth 1100*l*. a year to him, in order to devote himself to the conversion of the North American savages, by means of a college to be erected in Bermuda. The scheme was abandoned in consequence of the government dishonourably failing to perform its promises of aid; and Berkeley returned to England, after spending on the other side of the Atlantic seven years of his life, and the greater part of his fortune, in vain. Some of his biographers say that he had previously rejected an English mitre. After his return, however, he was made Bishop of Cloyne. He afterwards declined the see of Clogher, which was worth twice as much; and though urged by some of his friends, in 1747, to entertain thoughts of the vacant primacy of Armagh, he wholly rejected the idea. On removing to Oxford, in 1752, to superintend the education of one of his sons, he wished to resign the bishopric of Cloyne; but the king declared he should "die a bishop in spite of himself." He died in 1753.

The idealism of Berkeley, unlike that of the Germans, stands forth in the philosophy of the country which gave it birth almost as an insulated phenomenon—not as a normal development of principles before admitted, or regarded as established in a reigning school of metaphysics. It is quite at variance with the general sense and tendency of British thinking, whatever Berkeley may say to the contrary. For he persuaded himself that because the vulgar think only and talk only of what they actually see, hear, feel, taste, and smell, and never trouble themselves about any unknown *substratum* in which the qualities of the objects that occasion their sensations are supposed to inhere—they therefore are the abettors of his views in rejecting all materialism. "Rejecting all materialism"—an ambiguous expression this, it may be said. It may be so, and no doubt is so; but Berkeley tells us in brief what he means by it when he says that the "sun, moon, and stars are only so many sensations in their [men's] minds, which have no other existence but barely being perceived."* Whatever be the case in the Continental schools, and especially in Germany, certain it is that no considerable number of men in our country have ever maintained a doctrine which can be regarded as akin to this. We have as yet had no school of idealists. The development of the continental idealism, on the contrary, has

* "Principles of Human Knowledge," 94.

been gradual and continuous, from its germ in Descartes, through Malebranche, Spinoza, the Leibnitz-Wolfian school, and Kant, down to the strange and pantheistic phases which it has successively assumed in Fichte, Schelling, and Hegel. *We* have had no such development; and we are too cautious a people to be led away so far from *terra firma* and "common sense," (so much decried in some quarters) as to allow either our imagination or our logic to carry us, to such an extent, into the inconsistencies and dangers of intangible and airy speculation. Happily we prefer doubt, or even ignorance, to floundering, like the later Germans, beyond our depth in a sea which has neither a bottom nor a shore. And what is more—we are not ashamed to confess our ignorance or our doubt. This is the reason why we have not had among us what can properly be called a *school* of idealists—a school, we mean, that has with Berkeley maintained that mind or spirit is the only substance in the universe. For even Reid and Stewart were decided dualists, whatever interpretation akin to Kantism or even to Fichteism some of their statements may be regarded as capable of bearing, when exposed to a refined criticism which they never anticipated. A proof of this is contained in the words of Sir W. Hamilton, in a foot-note to Reid's Section on a "Material World;" as follows:—

"Consciousness assures us that we are immediately cognisant not only of a *self*, but of a *not-self*, not only of *mind* but of *matter;* and matter cannot be known as existing except as something extended. To this I venture a step beyond Reid and Stewart; though I am convinced that their philosophy tended to this conclusion, which is in fact the common sense of mankind."

Nevertheless, though Berkeley founded no school among us, and represented no school; it must be admitted that he has not stood entirely alone, in his denial of an external universe in the sense in which its existence is ordinarily maintained. John Norris, a clergyman, published his "Essay towards the Theory of the Ideal World," in 1704; though it does not appear that Berkeley was acquainted with this book. Its purport was to carry out the principle of Malebranche, *nous voyons tout en Dieu*, to its legitimate results. Malebranche said he could not deny a material world, because it appeared to him that the Mosaic account of the creation demanded its admission; but he did not know what use to make of it when he had admitted it; for he asserted that we have nothing to do with it, all our perceptions and ideas being the immediate effect of a sort of contact with the Deity; so that the states of our own minds are really attached to the mind of God. Norris does not appear to have cared what became of matter, his only concern being to establish that "all objects are seen or understood through the instrumentality of ideas; that these ideas do not derive their existence from the senses, but are part and parcel of the divine nature itself; so that an intelligible, that is ideal world exists really and only in God." Arthur Collier, Rector of Langford Magna, holds opinions in his "Clavis Universalis," published in 1713, completely identical with those of Berkeley, of whose speculations (which came out about the same time) he seems not to have been aware; though he had read Malebranche and Norris. Collier's mode of stating his argument is quite as clear and able as that of Berkeley; while as a writer he is not equal to the bishop in beauty of style and variety of illustration. It is worthy to be noted that, in some cases, he puts his argument almost in the same terms as Berkeley does.

"I declare that in affirming that there is no external world, I make no doubt or question of the existence of bodies, or whether the bodies which are seen exist or not—my inquiry is not concerning the existence, but altogether concerning the extra-existence of certain things or objects; or in other words, what I affirm or contend for, is not that bodies do not exist; but that such and such bodies, which are supposed to exist, do not exist *externally;* or, in universal terms, that there is no such thing as an external world."*

This is like Berkeley himself speaking, and he could not in the same brief space have more directly and guardedly stated the theory.

The inspiration which prompted Berkeley's zeal in contending for his idealism, was the conviction he entertained that the doctrine of materialism in all its forms, from the ancient atomic atheism to the dualistic doctrine of the co-ordinate existence of matter and spirit, was fraught with mischief to religion. He fancied that by banishing matter from the universe he should go far towards banishing atheism itself. For says he,—

"So great a difficulty hath it been thought to conceive matter produced out of nothing, that the most celebrated among the ancient philosophers, even of those who maintained the being of a God, have thought matter to be uncreated, and co-eternal with him. How great a friend material substance hath been to atheists in all ages, were needless to relate. All their monstrous systems have so visible and necessary a dependence on it, that when this corner-stone is once removed, the whole fabric cannot choose but fall to the ground; insomuch that it is no longer worth while to bestow a particular consideration on the absurdities of every wretched sect of atheists." He adds that, "men of better principles, observing the enemies of religion lay so great a stress on *unthinking matter*, should rejoice to see them driven from their only fortress, without which your Epicureans,

* "Clavis Universalis," pp. 3, 4.

Hobbists, and the like, have not even a shadow of a pretence, but become the most cheap and easy triumph in the world."*

What would Berkeley have said of the pantheistic idealism, of various phases, which was destined to be developed from the ideal side of the Kantian metaphysic?—developments which we know Kant himself would indignantly have rejected: but how far were some of these developments from theoretic atheism, and in what respect would Berkeley have regarded them as preferable to that of the pantheistic materialism?

Our author was even yet more sanguine in his anticipations of the good effects which were to arise from his speculations, if he could only establish them in the minds of men. He thought not only that matter as ordinarily believed to exist was the grand prop of atheism; he regarded it also as one great source of scepticism in respect to Christianity.

"For example, about the *resurrection*, how many scruples and objections have been raised! But do not the most plausible of them depend on the supposition that a body is denominated the *same*, with regard not to the form or that which is perceived by sense, but the material substance which remains the same under various forms? Take away this, about the identity of which all the dispute is, and mean by *body* what every plain ordinary person means, to wit, that which is immediately seen and felt, which is only a combination of sensible qualities or ideas; and then their unanswerable objections come to nothing."†

Our philosopher was especially desirous that his system might be clearly distinguished from that of Malebranche; and as a passage in which he points out the difference is at the same time explanatory of his own views, we will give it from the second of his three dialogues between "Hylas and Philonous," the work which contains the liveliest if not the clearest exposition of his views. We scarcely need premise that *Hylas* represents the ordinary cosmothetic materialism, as held by mankind in general; for so we venture to say, though our author maintains the contrary opinion: *Philonous* represents Berkeley himself and his system.

"I shall not be surprised if some men imagine that I run into the enthusiasm of Malebranche, though in truth I am very remote from it. He builds on the most abstract general ideas, which I entirely disclaim. He asserts an absolute external world, which I deny. He maintains that we are deceived by our senses, and know not the real natures or the true forms and figures of extended beings; of all which I hold the direct contrary, so that upon the whole there are no

* "Principles of Human Knowledge," 92, 93.

† "Principles," 95.

principles more fundamentally opposed than his and mine. It must be owned that I entirely agree with what the Holy Scripture saith, that in God we live and move and have our being: but that we see things in his essence after the manner above set forth, I am far from believing. Take here my brief meaning: It is evident that the things I perceive are my own ideas, and that no idea can exist unless it be in a *mind*. Nor is it less plain that these ideas or things by me perceived, either themselves or their archetypes, exist independently of my mind, since I know myself not to be their author; it being out of my power to determine at pleasure what particular ideas I shall be affected with upon opening my eyes or ears. They must therefore exist in some other mind, whose will it is they should be exhibited to me. The things, I say, immediately perceived, are ideas or sensations, call them which you will. But how can any idea or sensation exist in, or be produced by, anything but a mind or spirit? This indeed is inconceivable; and to assert that which is inconceivable is to talk nonsense: is it not?"

Thus anxious was Berkeley that his system might not be confounded with Malebranche's, which evidently approached to some of the speculations of the latter Platonists; though there is a still closer resemblance between Malebranche's "Vision in God" and the idealism of some of the Hindus; who, according to Sir William Jones, believed that the whole creation was not so much a work, as an *energy*, by which the Infinite Mind exhibits to his creatures a "set of perceptions, like a wonderful picture, or piece of music, always varied, yet always uniform." In a letter from Paris, in 1713, addressed to the patriotic and philanthropic Thomas Prior, Berkeley says: "I intend, to-morrow, to visit Father Malebranche, and discourse with him on certain points:" this interview took place, though it is not recorded in Berkeley's biography.* In the "Biographia Britannica,"† however, we learn that the question turned, as might be supposed, on the existence of matter; which though Malebranche contended for, he made no use of in his system. Disputes are said to be vehement, often, in proportion as parties come near together on controverted points, but do not coincide. Father Malebranche, who was now upwards of eighty years of age, was suffering at the time from inflammation of the lungs; and Berkeley found him in his cell preparing something for himself, and cooking it in a small pipkin. Unfortunately the aged father waxed very warm in the dispute about the existence or non-existence of a material world; and he so violently exerted his voice that he greatly increased his disorder, which carried him off in a few days. Dugald Stewart appears much to have relished the story of this

* By his brother, Dr. Robert Berkeley, and Dr. Stock.

† Vol. ii. p. 251.

philosophical rencontre, tragical as was its issue, and regrets that Berkeley did not make it the subject of a dialogue, like those between "Hylas and Philonous." "Fine as was his imagination," adds Stewart, "it could scarcely have added to the picturesque effect of the real scene."

After all, however, some of Berkeley's statements are so much like a description of Malebranche's system, that (bating the inconsequence of the latter in admitting a materialism which it made no use of, and which Berkeley denied,) it might almost be asked by one impatient of metaphysics, what difference there was, as to the actual perceptions of mankind, between "*tweedle-dum, and tweedle-dee.*" At all events there was one point of nearer approximation than Berkeley would seem willing to admit. For while Malebranche held that matter has no power to affect mind, that the ideas of all things exist in the mind of the Creator, and that we see all in Him, and He is our "intelligible world;" Berkeley, in his Third Dialogue, makes Philonous say to Hylas:

"When I deny sensible things an existence *out* of the mind, I do not mean my mind in particular, but all minds. Now it is plain they have an existence exterior to *my* mind, because I find them by experience to be independent of it. There is therefore some other mind wherein they exist during the intervals between the times of my perceiving them, as likewise they did before my birth, and would do after my supposed annihilation. And as the same is to me with regard to all other finite created minds, it necessarily follows that there is an omnipresent, eternal mind, which knows and comprehends all things, and exhibits them to my view in such a manner and according to such rules as he hath himself ordained."

The latter part of the above quotation, which represents the Deity as exhibiting sensible things to our minds, (taken in connection with what precedes,) might almost have been penned by Malebranche himself.

Kant describes Berkeley's idealism nearly in the same terms which the latter applies to that of Malebranche. The German metaphysician says that the name which he has given to his own theory (Transcendental Idealism, as founded on *à priori* or strictly primary and axiomatical principles,) will not justify any one in confounding it either with the empirical idealism of Descartes, who tried to doubt of everything before proof, excepting his own existence which he found it impossible to question—or with the "mystical and fanatical Idealism of Berkeley, and other chimeras of men's brains." Indeed Kant constantly maintains that it never entered his mind to doubt of the existence of things in themselves (*ding* an sich) or *material* objects; he merely denies that the sensuous representations of things (phenomena) *are* things in themselves. Kant charges Berkeley with an idealism which transforms real *things* into mere representations: on the other hand, the common notion of mankind with respect to the objects of our perceptions, which Kant says exalts mere *representations* into things —he chooses to call by the name of *visionary* (träumenden) idealism. Kant concludes by preferring the term *critical* to the term transcendental as a designation of his own idealism, which he regarded as the legitimate result of that self-review of the cognitive faculty which he proposed.* It is almost superfluous to add that Berkeley's scheme has nothing whatever in common with the subsequent development of idealism—Fichte's for instance; which was a subjective, egoistic, pantheistic hypothesis, in which the mind unconsciously created its own objects, (though it is remarkable that Berkeley states the notion of such an idealism in contrast with his own) or Schelling's spiritualized form of Spinozism—or Hegel's absolute idealism of thought, process, and relation.

Such is the licence and ambiguity of language, that we have almost as many meanings of the term *materialism* in the writings of philosophers, as of the contrasted term idealism. In its highest sense materialism involvês the entire rejection of all spiritual existence, as in the school of the ancient atomic atheism, and in that which marked the close of the eighteenth century in France. But we find the term used with great limitations; as among ourselves, for instance, in reference to the opinions of Priestley and others, who have denied the existence of mind or soul as a separate principle in man from the body, while they admitted a creating Spirit. Hartley's system of vibrations, again, is frequently denominated by the term "materialism;" in consequence of his attempting to account, in his mechanical way, not only for our sensations and emotions, but also for our associations, our most abstract ideas, and in short all our mental processes, whatever, even to the avowed rejection of Locke's second source of knowledge, namely reflection; which Hartley says is "not a distinct source," since "all the most complex ideas arise from sensation." Yet Hartley was very solicitous to obviate the inference that he held any materialistic notions with regard to the nature or essence of mind.

* Denn dass ich selbst dieser meiner Theorie den Namen eines transscendentalen Idealism gegeben habe, kann Keinen berechtigen ihn mit dem empirischen Idealism des Cartes, oder mit dem mystischen und schwärmerischon des Berkeley zu verwechseln. *u. s. w.—Prolegom.* (Rosenkranz, S. 51).

Wenn es aber ein in der That verwerflicher Idealism (Berkeley's) ist, wirkliche Sachen (nicht Erscheinungen) in blosse Vorstellungen zu verwandeln, mit welchem Namen will man denjenigen benennen, der umgekerht blosse Vorstellungen zu Sachen macht? Ich denke, man könne ihn den träumenden Idealism nennen, zu Unterschiede von dem vorigen der der schwärmende heissen mag. *u. s. w.—Ibid.*

The ordinary notion of matter has been that of something composed of separate resisting atoms, each having a distinct existence; there is, however, a view of it which may be termed the *dynamic*, in distinction from the atomic theory of it. To omit any ancient speculations, Leibnitz did not say that matter was a substance, but a *phenomène bien fondé;* sometimes he uses the participle or adjective *substantiatum* for it; but his meaning is more clear when he speaks of the "monads" of the lowest order (so called material atoms) as nothing but a kind of "*force.*" Boscovich, in 1758, advocated a dynamical theory, maintaining that the ultimate elements of things are unextended, or are in other words *mathematical points*, endowed with certain powers of attraction and repulsion; and that it is from these powers that all the physical phenomena of the universe arise. Now Berkeley's view of material objects was wholly opposed to all the above senses of materialism, the last equally with any of those which precede. Berkeley said, indeed, as we shall see, that he did admit the existence of material objects; but then we must interpret this assertion so as to make it harmonize with his total denial that there are any independent existences in the universe excepting spirits.

Previously to some further inquiry into Berkeley's main system, it is worth while to advert to his "New Theory of Vision," the publication of which preceded that of his "Dialogues." This work brought out into clear light a grand discovery in mental philosophy, and it exhibits much originality in the author. It had been concluded by philosophers as well as by mankind, that the cognisance which we take of the distances, figures, magnitudes, situations, etc., of objects, was the direct and immediate result of the power of vision. Berkeley was the first to establish to all future time, by a clear line of demarcation, the distinction between the *original* and the *acquired perceptions* of sight—to teach, indeed, the "art of seeing things which are invisible," as Dr. Reid has not unhappily expressed it. Berkeley clearly proved that distance, magnitude, position, and solidity, are not strictly to be called *visible;* that is, they are not the true and immediate objects of sight. By sight we see only coloured light; all the rest we learn solely by custom and experience. We learn to see just as we learn to speak and to read, only that we learn it more easily. On account of the instantaneous and almost uniform judgments which we very early form of the above-named affections of objects, we are induced to suppose that we have only to open our eyes, and thus to solve the whole mystery of vision: but we are deceived; we require the aid of our other senses. If we had only the sense of sight, we should have no means of determining anything but colour.

We have no proof that the true province of vision, as distinguished from that of the other senses, was known to any of the ancient metaphysicians. It is remarkable that Aristotle himself, by far the greatest philosopher of antiquity, has actually particularized the senses of seeing and hearing as examples of faculties which do not depend on custom or habit in their exercise, but give us immediate knowledge—making no distinction between what is direct and natural, and what is so obviously acquired, in these perceptions.* It is more surprising that Condillac, one of the most ingenious and popular of the French metaphysicians of the eighteenth century, and who had studied Berkeley's "Theory of Vision," should have argued at length, in his "Essai sur l'Origine des Connaissances Humaines," against the English doctrine of the acquired perceptions of sight; affirming in so many words that "the eye judges *naturally* of figures, of magnitudes, of situations, and of distances,"† and this, forty years after the publication of Berkeley's work. It is but fair to add that Condillac was afterwards convinced of his error, and expressly retracted it. It is perhaps still more singular that an attempt was made by an ingenious writer,‡ not many years ago, to prove the unsoundness of Berkeley's theory of vision—we hardly need say, as appears to us, with entire want of success.

Berkeley's psychological views on this subject have now long since been incorporated into the elements of optical science; and they were strongly corroborated, within twenty years of their being first promulgated, by the case of a youth who had been blind from infancy, who was operated on for cataract and restored to sight by the eminent surgeon Mr. Cheselden. The patient felt that everything was in his eye at once — of distance he could form no judgment till he had learnt it by experience. He knew a dog from a cat by feeling, during his blindness; but when couched he had to form the associations between feeling and sight, before he could distinguish them with his eyes open. At first he did not know by sight the shape of anything, nor could he distinguish magnitudes in this way. In short, Berkeley's theory was entirely established; and subsequent cases of couching, which have sometimes been put forth as opposing it, have when fairly examined been admitted to agree with it.

Though no one had ever before pursued the true theory of vision, as Berkeley did, to the extent of marking a new epoch in psychology and optics—it must not be supposed that in his hands the theory was in all its elements original: indeed he did not himself claim that it should be so considered; but rather that it was partly a correction, partly an extension and completion of principles which had been

* "Ethic. Nicomach," lib. ii. cap. 1. † Sect. vi.

‡ "A Review of Berkeley's Theory of Vision;" by S. Bailey, 1842.

partially admitted or hinted by previous philosophers. The "Optics" of Alhazen, and the "Optica Promota" of James Gregory, among other writings on the same subject, may be named as examples. Malebranche also had clearly anticipated some of the metaphysical bearings of the subject. Nor must it be forgotten that our immortal Locke himself had already shown his remarkable sagacity in anticipating the fact, as proved by Cheselden's patient, that a blind man when first restored to sight would not know a "cube" from an object of any other figure. Indeed Locke had been even more explicit: he says, respecting perception, that "the ideas we receive by sensation are often altered by the judgment, without our taking notice of it." He instances a globe, which when before us presents to our minds the "idea of a flat circle variously shadowed," while "our judgment by habitual custom frames to itself the perception of a convex figure." He also alludes to painting as illustrating the same thing. He adds: "Space, figure, and motion, by their several varieties, change the appearances of light and colours which are the proper objects of sight; so that we bring ourselves by use to judge of the one by the other. This, in many cases, by a settled habit of things whereof we have frequent experience, is performed so constantly and so quick, that we take that for the perception of our sensation, which is an idea formed by our judgment; so that one, viz. that of sensation, serves only to excite the other, and is scarce taken notice of itself."* From this language of Locke, it is clear that he really did, in the main, anticipate the very same conclusion respecting the effect of association and habit on our perceptions of sight, which Berkeley developed in detail; and which renders his "Theory of Vision" so valuable a contribution to human knowledge, and especially to mental philosophy—we may say the best of his contributions.

It is, however, his psychological theory—in fact his reduction of the whole universe to a psychology, that has distinguished him as one of the acutest and boldest, if not most satisfactory of thinkers. His theory may be equally well learned from his "Principles," or his "Three Dialogues." In the former, however, there is greater condensation; while the latter are by far the more lively and amusing. His learning in the history of philosophy is but little shown in these works: he seldom mentions the names either of ancients or moderns; but originality of thought and illustration everywhere abounds. Alarmed at the irreligious and atheistic tendencies which he saw threatening, in his day, and thinking that they depended for support mainly on the prevailing notions about *matter*, he was led to inquire into the

* "Locke's Essay;" book ii. chap. 9, § 8, 9.

claim which these notions had to our belief, and finally to reject them altogether. Materialism he held to be not only the chief "ground of scepticism, atheism, and irreligion;" but at the same time the "chief cause of error and difficulty in the sciences." With regard to religion both natural and revealed, Berkeley was somewhat visionary in his expectations of the benefits which would arise, if immaterialism could but prevail. He thought that if the common theory of matter were once banished, much would be done for Christianity, and against certain forms of unbelief.* But how closely blended with unbelief has been the idealism which has been developed in the third period of the German school of philosophy! We fancy that Berkeley would not have found some of these idealistic theories a whit more to his mind, as to their religious bearing, than the materialism of which he had so characteristic a horror; and would have acknowledged, had he been witness of their rise and progress, that it must take something more than the overthrow of all materialism to destroy unbelief, and to regenerate the world.

Berkeley's views, though minutely unfolded and at great length in his works above-named, are more capable perhaps of being condensed within a small compass than most philosophical theories. We shall endeavour to give as brief a compend of them as possible.

He holds that the opinion that we have a power of framing *abstract ideas* is to be specially deprecated,† as having led men to these notions about a material universe. He admits that we have "general ideas," but not "*general abstract ideas;*" which we certainly have not, in the sense Berkeley intends (evidently that of the scholastic Conceptualists); for undoubtedly we cannot frame in imagination the picture of a triangle which is of no species, and yet of all, at once, as Locke describes, not very happily, in his Essay:‡ but we can readily think of some quality in which all triangles agree; and we can use one, therefore, as the representative of all. Having fairly demolished abstract ideas, in the above unintelligible signification of a sensuous form or *schema* including various species, our philosopher considered that he had given a fatal blow to the doctrine of a *substratum*, or support of sensible qualities or phenomena, such as matter is held to be. These sensible qualities, he maintained (as being only in us), require no synthesis or bond to unite and sustain them,—all such synthesis is merely the invention of our own imagination; it is purely mental.

Locke had maintained that all our knowledge consists in the recognition of the relations of our ideas, not marking the distinction between logical and psychological judgment, though admitting the latter

* "Principles," 95, 96.

† *Ibid.* Introd.

clearly enough under the name of intuition. Ideas, then, are the true objects of knowledge. Very right, said Berkeley, according to the ancient Platonic doctrine; and he added: "It is evident that the objects of human knowledge are either ideas imprinted on the senses [sensations], or such as are perceived by attending to the passions and operations of the mind; or lastly, ideas formed, by help of memory and imagination, from those originally perceived in the aforesaid ways."* It is evidently with the first kind of ideas that Berkeley's theory has mainly to do. Thus, for instance, a certain well-known collection of our ideas which have always been found connected, are signified by the name *apple;* other collections may be a tree, a book, etc., respectively. Now these ideas can only exist in a mind which perceives them, and the existence of our minds is here assumed on the alleged testimony of consciousness. And as all allow that our passions and fictions of imagination do not exist externally to the mind, so it is not less evident, says our author, that "the various sensations or ideas imprinted on the sense, cannot exist otherwise than in a mind perceiving them." This Berkeley regards as intuitively proving that the objects which we call material are nothing more nor less than sensible ideas, or sensations. "The table I write on exists, that is, I see and feel it; and if I were out of my study I should say it existed, meaning thereby that if I was in my study I might perceive it, or that some other spirit actually does perceive it. For as to what is said of the absolute existence of unthinking things, without any relation to their being perceived, that seems perfectly unintelligible. Their *esse* is *percipi,* nor is it possible they should have any existence out of the minds of thinking beings which perceive them."† "It is indeed an opinion strangely prevailing amongst men, that houses, mountains, rivers, and, in a word, all visible objects, have an existence natural or real, distinct from their being perceived by the understanding. Yet whoever shall find in his heart to call it in question may, if I mistake not, perceive it to involve a manifest contradiction. For what are the forementioned objects but the things we perceive by sense, and what do we perceive besides our own ideas or sensations?"

Now, says our author, since the objects of sense, which are my own ideas (sensations) when I am recognizing them, evidently exist when I am absent from them, they have a real existence; and as they can only exist in a mind, there must be some other spirit wherein they exist, in the intervals between my perceiving them; there is therefore an infinite, omnipresent Spirit who contai[illegible]s and supports them. This is Berkeley's *main argument* for the Divine existence, and he holds it

* "Principles," 1. † Ibid. 3, 4.

to be irresistible. Philonous says to Hylas, in the Second Dialogue that he differs from the philosophers who say, "There is a God, therefore he perceives all things;" whereas, in order to state the whole case, they ought to say, "Sensible things do really exist, and are necessarily perceived by an infinite mind; therefore there is an infinite mind, or God." Philonous adds, "This furnishes you with a direct and immediate demonstration, from a most evident principle, of the being of a God. Divines and philosophers had proved beyond all controversy, from the usefulness and beauty of the creation, that it was the workmanship of God. But that an infinite mind should be necessarily inferred from the bare existence of the sensible world, is an advantage to those only who have made this easy reflection; that the sensible world is that which we perceive by our several senses; and that nothing is perceived by the senses beside ideas; and that no idea or archetype of an idea can exist otherwise than in a mind." Thus does Berkeley rest the chief argument for the being of God on the assumption that the objects of nature are neither more nor less than our perceptions of sense; which objects are nothing but as perceived; so that, as they are not always perceived by man, they must have an omnipresent mind to exist in. Elsewhere, however, it is fair to say, Berkeley dwells eloquently on the argument from causation; there are agencies at work producing in us the ideas which are the actual objects of nature—what are these agencies?—the perpetual actings of the Creator.

In support of his theory of the utter inconceivableness of material things as substances, Berkeley (alluding probably to Locke's statement that "the ideas of the primary qualities of bodies are resemblances of them, and their patterns do really exist in the bodies themselves"*) maintains that ideas can only resemble ideas, which is strictly true; but he need not, on this account, have denied that external causes may be adapted, we know not how, to excite in us certain sensations and ideas. Again: as we "only know by sense and reason, and as neither informs us of the unperceived material *substratum*, but only of our sensations and ideas," says our author, even if there *were* solid bodies without our minds, we could never know the fact. That we may have all the ideas of matter which we now have, in "dreams and phrensies," is granted by all, when there is no matter causing them, so that at all events matter is useless, since we can have all our ideas without it.†

In justice to Berkeley we must say that there is nothing which he more insists on than that, on his principles, "each part of the mundane system is as much a *real being* as on any others." He must mean, how-

* "Essay," ii. 8. † "Principles," 8, 9, 10, 18.

ever, that the actual operations of the laws of nature, that is, the constant agencies of the Creator, are real facts occurring in the consciousness of minds. He can or ought to mean nothing more; for he constantly asserts that our own subjective states (sensations) *are* the objects around us. He allows to spirits the name of substances, and to them alone. On this point he is very decided, talking of spiritual *substance* as something beyond its attributes or qualities, just as most men talk of *matter* as something beyond the properties which belong to it. He remarks that no idea can be formed of such a spirit; for "all ideas being passive and inert, they cannot represent to us, by way of image or likeness, that which acts." His language, however, respecting spirit, is frequently obscure. It occasionally seems idealistic even in the most absolute sense of the Germans: for he sometimes perfectly identifies understanding and will with soul and spirit. But it is evident, on the whole, that he means to admit some being that is distinct from its own acts, and is called spirit; for he says, "it must be owned that we have some *notion* of soul," and its operations; and he speaks of it as an agent, and as the only substance or support wherein unthinking beings or ideas can exist. Soul, spirit, and substance mean a "real thing," not an idea. "What I denote by the term *I*, is soul or spiritual substance." It is an active being, the existence of which consists in perceiving ideas and thinking."* This last statement, again, (such is the fluctuation of Berkeley's language,) might readily be construed into absolute idealism, in the later German sense: and there are other passages of the same kind; in one at least of which our author speaks, in an apparently slighting manner, of the common distinction between will and understanding, and a substance supporting these powers. In fact Berkeley is not always consistent with himself, and this is an example: for he constantly applies the term substance to mind, while he stoutly and uniformly denies it to everything else. Charity, therefore, must conclude that he did not mean to imply, with Hume, that sensations and ideas are the only things in the universe; or, in other words, that God and created spirits are mere conventional terms, amounting to nonentities; for he expressly excludes them from being "ideas;" and speaks of them as "active, simple, uncompounded substance, which cannot possibly be affected by the decays which befal natural bodies."†

Berkeley's hypothesis produced no inconsiderable noise in the reading world when it first became known; and it was just the kind of thing to furnish a very cheap and easy theme for ridicule. Arbuthnot wrote to Swift: "Poor Berkeley hath now the idea of health; which was

* "Principles," 27, 135, 139. † Ibid. 14.

very hard to produce in him, for he had an idea of a strange fever on him." The most absurd thing of all was Beattie's angry declaration that if these principles prevailed, they "would soon issue in the extermination of the human race!" Berkeley's doctrines, however, were adopted by Bishops Sherlock and Smallridge; and afterwards by Drummond and Kirwan. Dr. Reid, whose criticisms of Berkeley are not always very analytical, discriminating, or candid, admits that he at one time fully believed his doctrine. The following is the way in which Berkeley was wont to reply to some of the most ludicrous or popular objections which have been made to his views—objections such as the above, and which we would strongly recommend to the adoption of all superficial readers of a little philosophy, who are solicitous to show their wit.

"You say, it sounds very harsh to say we *eat and drink ideas*, and are *clothed with ideas*. I acknowledge it does so, the word idea not being used in common discourse to signify the several combinations of sensible qualities which are called *things;* and it is certain that any expression which varies from the familiar use of language will seem harsh and ridiculous. But this doth not concern the truth of the proposition, which is no more than to say, we are fed and clothed with those things which we perceive immediately by our senses. If, therefore, you agree with me that we eat and drink, and are clad with the immediate objects of sense which cannot exist unperceived or without the mind, I shall readily grant it is more proper or conformable to custom that they should be called things rather than ideas."*

Hylas, in the Third Dialogue, asks whether it does not follow from the principles laid down, that "no two men can see the same thing? and is not this highly absurd?" Philonous replies: "If the term *same* be taken in the vulgar acceptation, it is certain (and not at all repugnant to the principles I maintain) that different persons may perceive the same thing. Words are of arbitrary imposition." Berkeley then enlarges on the ambiguity of the word *same;* and says that "same" may be very well applied to "agreement in perceptions: the dispute is about a word. I know not what you mean by the *abstracted idea of identity;* and should desire you to look into your own thoughts, and be sure you understand yourself, Hylas. Take this further reflection with you: the materialists themselves acknowledge what we immediately perceive by our senses to be our own ideas. Your difficulty, therefore, that no two see the same thing, makes equally against the materialists." Thus did our author dispassionately show that if his system was to be confuted at all, it must be by some other method than popular objections and claptrap ratiocinations; and

* "Principles," 38.

thus dexterously did he avail himself of the *argumentum ad hominem* in allusion to the theory so long current in the schools—that all our knowledge can only be of our own ideas.

Berkeley's whole argument reduces itself to two aspects: one is, that we have no evidence of what is called a material world; all that we know may be given to us without it: and even if there were such a world we never could know it; for whatever knowledge we have of outward things must be by the senses, which can only give us knowledge of our own sensations; and these sensations, being affections of mind, can have no resemblance to a thing which is unthinking and inert. The other point of view regards Berkeley's assertion of the impossibility and absurdity of a material world; for he pronounces most confidently that "the existence of bodies out of a mind perceiving them is impossible, and a contradiction in terms."*

The latter and most dogmatical part of Berkeley's theory, is entirely destitute even of plausibility; and appears to us opposed to what might have been expected from his devoutly religious character. For who shall set limits to the Divine Omnipotence! Of course, logical contradictions involve impossibilities; and they express what cannot be conceived of as having any relation to the Divine power: as, for example, that a square may have *five sides;* or that a triangle may be constructed which has the sum of its angles equal to *three right angles.* But where is the logical contradiction of supposing a material world? If it be in the power of God to produce all our present sensations and ideas by his own immediate agency, without the intervention of any *third* existence of any kind between us and Him; and to do this in such a way as that we shall always be irresistibly, though by an illusion, led to believe in this *tertium quid*—something different both from Himself and from ourselves, and from our own bare sensations or ideas, and which immediately causes them—(and that it is quite conceivable that the Deity might do so if it pleased Him, we are far from denying:) then, on the other hand, what earthly or heavenly reason can be assigned—why it cannot be regarded as a possible exertion of the Divine power that the Creator should, amidst his infinite resources, form an existence that should not have the attributes of thought, feeling, and will, as *mind* or spirit has—but which should have a totally different set of attributes, by means of which sensations and ideas should be produced in us? And if it be asked how could anything but spirit act on spirit?—the retort is ready, how can or does spirit act on spirit? We know not how. It does, certainly, appear to us to require a little more boldness than is worthy of a cautious

* Second Dialogue.

inquirer after truth, to assert that it would be a logical absurdity to suppose that God may choose to deposit in an unthinking existence (matter) certain forces which shall act on us, according to what we term the laws of nature. Yes, undoubtedly matter *may* exist—it is not an impossibility or an absurdity, at all events; and if we were very eager to saddle men's theology with the vices of their metaphysics, we should be disposed to say that to pronounce "unthinking substance impossible" is next-door to presumption. But we would rather call it a violent assumption; or if we regard it as a conclusion —a conclusion resting on nothing but assumptions for premises.

The other part of Berkeley's speculations, in which he virtually challenges his opponents to *prove* the existence of a material universe is, of course, less easily capable of being dealt with. For how can we prove what seems to present itself to our faith, every moment, with such direct and commanding self-evidence as to defy and supersede all proof—a self-evidence second only to that of our own thinking existence? It is enough, however, if we are right in saying that Berkeley, with all his acuteness and aptness for the subtilties of metaphysics, has not one whit shaken the doctrine of a material world: he has left the question just where he found it. His whole theory is based on *assumptions* from beginning to end—assumptions sometimes his own, sometimes adopted from the current philosophical opinions, or from his view of them—but assumptions still; and these assumptions are not seldom blended with inconsistencies and inconsequences.

It would be easy to bring forward germs and analogies of Berkeleianism among the ancient Greeks. Even in Plato we find the suggestion that it would be difficult to prove that the life of man is not a continued sleep, and that all our thoughts are anything but dreams. But among the Sophists and the Sceptics we have what is bolder than conjecture. Protagoras maintained that thought is sensation, and that all our knowledge is phenomenal. Berkeley himself cannot be excused from having dogmatically confounded sensations with ideas and perceptions. Protagoras also said that "man is the criterion of all that exists; all that is perceived by him exists; that which is perceived by no one does not exist."* This is a sort of Grecized Berkeleianism. Our philosopher had an example of cosmothetic doubting nearer home, in Descartes; who, however, finally anchored his belief of materialism in the "veracity of God."† Malebranche rejected this argument, in one sense, but adopted it in another; for he admitted the existence of matter only as what he conceived to be a

* Sextus Empiricus, "Hypotyp. Pyrrhon," 44.

† "Sixth Meditation." It is remarkable that Descartes put down, among the possibilities, a theory of idealism precisely identical with that of Fichte.

revealed truth. Berkeley, as we have already seen, regarded himself as authorized by Locke, and the current opinion of the day, to connect the theory of matter with the dogma that "the primary qualities of body are resemblances of our sensations." Now, on this latter assumption, we take it, the whole gist of that part of his argument in which he attempts the disproof of matter, may be said to lie. Probably the briefest form in which this doctrine can be put is the following conditional syllogism: "If matter exists, it must resemble the ideas which we have of it in our minds: but the properties of an unthinking being cannot resemble the ideas of a conscious mind; therefore matter does not exist."

Now we hold with Berkeley, that our ideas and sensations can only resemble our ideas and sensations: these modifications of a conscious being can only have a mutual resemblance. The idealistic theory of resemblance must, therefore, be abandoned. But when our philosopher asserts that if matter exists, it must thus resemble the idea we have of it in our minds—we regard his position as a sheer assumption. Matter cannot be inert and extended, he says, unless mind be inert and extended. Now this was saying that the cause must necessarily resemble the effect. This is an assumption which would involve the most absurd consequences. Does the energy of will, or of any nervous force which our will excites to action, to move our arm, resemble the motion itself? Does the Deity who immediately produces, according to Berkeley, all our sensations, resemble those sensations? Does the view of impending danger resemble the emotion of terror of which it is the cause? And is there not, moreover, in Berkeley's system a kind of confounding of cause with effect? We are told that sensible ideas (the only *things* in the world) are now in our *own* minds, now in the mind of God; who presents them in our consciousness according to his own laws. These magical ideas are, of course, effects when they exist in our minds; but we are told they have an existence in the mind of the "Eternal Spirit," when no created being is conscious of them—what then are these same things now but causes, causes which are to become effects when creatures are experiencing these sensible ideas?

Again: what strange consequences follow from Berkeley's distinct assertion that the sensible world has an existence independently of our minds; while at the same time he maintains that when I am pointing to an object, this object has no existence external to my mind! This table is my sensation, and yet this table (my sensation) can have and must have an existence out of me! Where, then, is now the sensation?—in the Deity, who is a spirit! There is nothing but mind, says our author, existing in the universe—all else is but a modification of mind : and yet the modifications of my mind have an *existence* apart from my mind. The fact is, that Berkeley's language is often liable to the most contradictory interpretations—interpretations which, of course, he would in his own way have sought to repudiate. We have too, in this system, it would seem, a kind of syncretism of inconsistent idealisms. While Berkeley would annihilate matter, he speaks of sensible objects in a way which is only due to things which are separate entities, and which in some inconceivable way may pass from one mind to another. These sensible ideas exist at this moment in my mind; but they will not do so when I am asleep. They exist *then*, elsewhere. But what are these existences, which can have, elsewhere, all the being which they have now in my own mind? They are, Berkeley tells us, not active things—they are passive and inert; and we have seen that their residence may be reciprocated from mind to mind. These statements cannot but suggest to the student of the history of philosophy some of the dogmas of the ancient atomic idealism of Leucippus, Democritus, and Epicurus, as we have it described by Lucretius, in his poem " De Rerum Naturâ"—a theory from which, of course, Berkeley recoiled with horror.

Again: Berkeley says that by our senses we have no knowledge of anything but our sensations and impressions of sense; and this is said with a view to get rid of all basis for any positive real being distinct from mind. Now, it should be remembered that while sense can only, in itself, convey to us sensations, our senses do not act alone, but in connexion with the intellectual faculty, which peremptorily tells us that they must have a cause; for to believe in causation is constitutional to the human mind. True, we cannot *prove* that every change must have a cause; but we cannot deny it—we cannot doubt it. Does not every rational being *know* that each event which happens in the universe around us, must have its cause? But what is the origin of this knowledge? The reply is : sense gives us changes, events, effects; but the intellectual faculty, on occasion of the experiences of sense, rises beyond experience to the necessary and universal truth that every change must have a cause, in all time, and in all worlds. Berkeley's principle, therefore, that sense can give us sensation alone, falls decidedly short when put forth as an argument against matter, however true it is in the letter.

Nor is it of any avail to our author to attempt to include the whole of our knowledge within the limits of our immediate consciousness. We are not concerned with the opinions respecting ideas which Berkeley either found, or supposed he found, current in his day—we limit our criticism to his own assertion that all the " objects of human knowledge are either ideas imprinted on the senses; or such as are perceived by attending to the passions and operations of the mind; or lastly, some combination of the above by memory and imagination." Now this doctrine, we should remember, is propounded with the view of upholding the principle that we cannot know matter, because our knowledge is limited to the modifications of our own consciousness; but, if so, then how can we know mind?—how can we know God? Berkeley constantly proceeds on the understanding that our knowledge does extend to the assurance of the existence of ourselves, of mind, and of a Deity. He distinctly says that mind is the only substance in the universe, and he speaks of its powers; but how, on his principles, is he entitled to this dogma? We do not perceive *self* as we perceive its modifications; we do not take direct cognisance of the substance mind, as we do of its attributes; we do not perceive God by any intuition, sensuous or intellectual, as we perceive his operations. Berkeley did not deny that all properties imply a substance: but he chose to assume that all properties are properties of mind. Hume saw that, on the principle that we can know nothing but our own ideas and "impressions" (sensations), we are not entitled to affirm any substances—either a created mind (not even a *me*) or a God. Mankind, says Hume, in his "Treatise of Human Nature," are only a "bundle of perceptions." According to him, mind is as much a mere synthesis of properties as matter is. We do not see that this deduction was anything more than the legitimate climax of that idealism which led Berkeley to reduce all the material universe to a mere synthesis of the imagination—a synthesis of sensations, not a substance possessing its own qualities and powers.

We maintain, therefore, in conclusion, that Berkeley's idealism is, after all, incompetent to the grand object he had in view, the establishment of a pure immaterialism on the ruins of matter. He as much assumes the existence of the human soul, as mankind in general assume that of the substance matter. He assumes a Deity as a necessary depository of ideas, even more than he assumes his necessity as a Creator. He argues against matter as an unperceived *noumenon*—he admits mind, a *noumenon* equally unperceived. If the material universe be only an idea of our own, why not also the spiritual? If it be replied, there must be causes—then how do we know them, since they are not our own consciousness, but only a rational suggestion, inference, conclusion, intuition—call it what we may.

We have only to repeat that Berkeley's whole argument against the existence of matter, ingenious and subtle as it is, and propounded with extraordinary ability, leaves the question just where it was before—just where the Greek sceptics left it, two thousand years ago, and more. The ultimate basis of human knowledge has always been assailed by scepticism, because it admits not from its very nature of being fortified by proof; and yet our most certain knowledge reposes on no other foundation than self-evident principles. What matter is, has long been a puzzle to natural philosophers; but even the dynamic theory of it, which goes the farthest way towards Berkeley's denial of its substantial existence, is far enough from the assumptions, the extravagances, and the final goal of his system. The machinery of this system, we are bold to say, instead of clearing up any difficulties and making everything plain, as the author supposed, has but introduced further grounds of scepticism. If the advance of the human mind out of the immediate sphere of its own subjective consciousness to *objective* knowledge is a puzzle, Berkeley has not solved it by confining matter wholly to the sphere of consciousness: he has only cut the Gordian knot, by an hypothesis which would prove too much even for his own ultimate purpose—he has not untied that knot.

---

